# Chemoradiation versus surgery for superficial esophageal squamous cell carcinoma after noncurative endoscopic submucosal dissection: comparison of long-term oncologic outcomes

**DOI:** 10.1186/s13014-022-02162-8

**Published:** 2022-11-19

**Authors:** Gen Suzuki, Hideya Yamazaki, Norihiro Aibe, Koji Masui, Takuya Kimoto, Shinsuke Nagasawa, Shou Watanabe, Shou Seri, Akito Asato, Atsushi Shiozaki, Hitoshi Fujiwara, Hirotaka Konishi, Osamu Dohi, Takeshi Ishikawa, Hany Elsaleh, Kei Yamada

**Affiliations:** 1 Departments of Radiology, Graduate School of Medical Science, Kyoto Prefectural University of Medicine, 465 Kajiicho Kawaramachi Hirokoji, Kamigyo-Ku, Kyoto, 602-8566 Japan; 2 Gastroenterology and Hepatology, Graduate School of Medical Science, Kyoto Prefectural University of Medicine, Kamigyo-Ku, Kyoto, Japan; 3 Digestive Surgery, Graduate School of Medical Science, Kyoto Prefectural University of Medicine, Kamigyo-Ku, Kyoto, Japan; 4 Department of Radiation Oncology, The Alfred, Melbourne, VIC Australia

**Keywords:** Esophagectomy, Esophageal neoplasms, Esophageal squamous cell carcinoma, Endoscopic mucosal resection, Disease-free survival, Chemoradiotherapy

## Abstract

**Background:**

Esophagectomy is the standard adjuvant treatment for superficial esophageal squamous cell carcinoma (SESCC) following noncurative endoscopic submucosal dissection (ESD). However, recent reports have also shown that ESD with adjuvant chemoradiotherapy (CRT) has promising results. This retrospective study aimed to elucidate the efficacy of CRT compared to surgery in patients with SESCC after noncurative ESD.

**Methods:**

This study retrospectively compared the long-term outcomes of patients who received adjuvant treatment with surgery or CRT after noncurative ESD for SESCC.

**Results:**

Data were collected from 60 patients who developed SESCC after noncurative ESD, 34 of whom received adjuvant chemoradiotherapy (CRT) and 26 underwent esophagectomy. The median follow-up periods were 46 and 56 months in the CRT and esophagectomy groups, respectively. The median patient age was significantly higher in the CRT group than in the esophagectomy group (69 vs. 65 years, *p* = 0.0054). CRT was completed in all patients, and the incidence of grade ≥ 3 nonhematologic adverse events was 6%. The overall and disease-free survival did not significantly differ between the two groups.

**Conclusions:**

CRT following ESD seems a promising nonsurgical strategy for optimizing the selection of therapies for high-risk SESCC and warrant further investigation.

## Background

Esophageal cancer is the sixth most common cause of cancer-related deaths globally, and therapy for esophageal cancer is based on the patient’s disease stage, age, and performance status [1]. Endoscopic submucosal dissection (ESD) is now one of the standard treatments for T1a esophageal tumor and is increasingly performed in patients with superficial esophageal squamous cell carcinoma (SESCC) due to its ability to remove shallow submucosal (T1b:SM1-2) tumor [2–4]. However, high-risk SESCC, such as tumors with a positive resection margin, muscularis mucosae invasion with lymphovascular invasion (LVI), or submucosal invasion, are usually not curable with ESD alone and require adjuvant treatment such as surgery or chemoradiotherapy (CRT) [5], and it is unclear whether surgical treatment or CRT is the optimal treatment choice after noncurative ESD. Esophagectomy with lymph node dissection is considered a standard treatment for high-risk SESCC. However, it has a number of drawbacks, such as a high risk of serious complications, risk of perioperative death, lengthy recovery period, and a potential for long-term dysphagia [6, 7]. Regardless of the clinical stage, esophageal cancer with poor surgical indications has been successfully treated with CRT, even in elderly patients [8]. Because ESD and CRT both enable organ preservation and are relatively less invasive than surgical resection, some researchers have suggested adding CRT instead of esophagectomy as an adjuvant treatment after ESD for SESCC [9–12]. However, CRT-related late toxicities can occasionally lead to death [10, 13, 14]. Previously, we directly compared the outcomes of esophagectomy and CRT using a reduced irradiation field to establish a safer and more effective adjuvant treatment after noncurative ESD. Our results showed that CRT was comparable to esophagectomy in terms of overall survival (OS) and disease-free survival (DFS), with acceptable side effects [12]. In this study, we examined a larger number of cases over a longer follow-up period than in our previous study to better define the role of adjuvant CRT after noncurative ESD for high-risk SESCC. To the best of our knowledge, this is the largest study directly comparing surgery and CRT after ESD for SESCC.

## Methods

### Patients

The study was approved by the institutional review board of the Kyoto Prefectural University of Medicine (Approval number ERB-C-1104). We analyzed patients who required adjuvant treatment (surgery/CRT) after ESD for SESCC from January 2008 to December 2021 at our institution. The indications for ESD in our study include as follows: (1) depth of tumor invasion is diagnosed as T1b (SM1-2) by endoscopy and endoscopic ultrasonography, (2) clinically node-negative (cN0) and no metastasis to other organs (cM0), (3) circularity of esophageal lumen is less than three-fourths and (4) no ulcerative lesion in the tumors. For the adjuvant CRT group, consecutive patients treated after January 2014 were included, as in our previous studies [12], to properly assess the outcome of a unified treatment strategy. Definitive adjuvant treatment was recommended for patients who had undergone noncurative ESD for submucosal or muscularis mucosae cancers with LVI and a positive resection margin [15, 16]. Written informed consent for ESD followed by adjuvant therapy was obtained from all patients.

### Process for deciding on adjuvant treatment

The standard adjuvant therapy after noncurative ESD for SESCC is esophagectomy with lymph node dissection. CRT is recognized as an alternative adjuvant therapy. Patients were assigned to surgery or CRT, as decided by the patient and their oncology team after they had received a full explanation from surgeons on the surgical aspects and medical oncologists on the CRT option. All patients were involved in the decision-making process to provide adjuvant treatments following ESD.

### Chemoradiotherapy

Megavoltage photon beam radiotherapy was concurrently initiated with systemic chemotherapy. All patients underwent computed tomography (CT) simulations before treatment. Before obtaining a planning CT scan, the tumor bed was endoscopically marked with a clip. The location of the tumor bed was defined based on the scarring tissue created by ESD. Three-dimensional conformal radiotherapy with a linear accelerator (6 or 10 MV) was applied to the treatment. A dose of 40 Gy in 20 fractions was administered to the initial clinical target volume (CTV1) in patients with negative resection margins to prevent lymph node recurrence. CTV1 included the regional nodal area as follows: (1) the cervical/upper thoracic esophagus, comprising the bilateral supraclavicular and mediastinal lymph node regions to the bifurcation of the trachea for upper esophageal cancers; (2) the middle thoracic esophagus, consisting of the superior mediastinum and 2 cm below the distal end of the tumor bed marked with a clip oriented along the esophagus; and (3) the lower thoracic region, involving the tumor bed with 2-cm craniocaudal margins oriented along the esophagus. For patients with a positive resection margin based on pathological diagnosis after ESD, additional 10 Gy boost irradiation to the tumor bed with 2-cm craniocaudal margins (CTV2) was applied. The planning target volume was defined as the CTV plus 1-cm margins in all directions in the initial and boost plans. Other details on radiotherapy have been previously described [12]. The chemotherapy regimen included continuous 5-fluorouracil (FU, 1000 mg/m^2^/d on days 1–4 and 29–32) and cisplatin (CDDP, 75 mg/m^2^/d on days 1 and 29). Two patients with heart failure used nedaplatin instead of cisplatin.

### Follow-up and evaluation

All patients were followed up to detect local recurrence or distant metastasis every 3–4 months during the first 2 years and every 6 months thereafter, with blood tests, upper gastrointestinal endoscopy with iodine staining, and CT of the neck/chest/abdomen. Follow-up data were obtained from the electronic medical records. Locoregional recurrence was defined as the recurrence of the primary tumor or metastases to the regional lymph node observed on endoscopy or CT.

### Statistical analysis

The baseline characteristics of treatment groups were compared using the Mann–Whitney *U* test for continuous variables and the χ^2^ test or Fisher’s exact test for categorical variables. OS and DFS were calculated using the Kaplan–Meier method. OS was assessed from the date of treatment initiation to the date of the last follow-up or death from any cause. DFS was assessed from the date of treatment initiation to the date of the first observation of any recurrence or death from any cause. Differences between the groups were estimated using the log-rank test. All statistical analyses were performed using EZR (Saitama Medical Center, Jichi Medical University, Saitama, Japan), a graphical user interface for R (The R Foundation for Statistical Computing, Vienna, Austria) and a modified version of the R commander designed to add statistical functions frequently used in biostatistics [17]. In all analyses, *p* < 0.05 was considered significant.

## Results

### Patient characteristics

Sixty patients were treated with ESD followed by esophagectomy or CRT. Thirty-four (57%) patients received adjuvant CRT (CRT group), while 26 (43%) received esophagectomy (esophagectomy group). The median observation period was 49 (range, 4–144) months (46 months in the CRT group and 56 months in the esophagectomy group), and the median age was 68 (range, 45–80) years. The median patient age was significantly higher in the CRT group than in the esophagectomy group (*p* = 0.0054). There were no significant differences between the two groups concerning sex, tumor location, depth of tumor invasion, LVI, and positivity of the endoscopic surgical margin. In the esophagectomy group, 3 patients underwent transthoracic esophagectomy, and 23 underwent esophagectomy via a laparoscopic transhiatal approach. All patients underwent two-field (thoracic and abdominal) lymphadenectomy. Post-surgical reconstruction was performed with a gastric tube via the post-sternal route. Table 1 summarizes the patients’ characteristics.

**Table 1:** Patient characteristics

Characteristic	All (n = 60)	Chemoradiation (n = 34)	Esophagectomy (n = 26)	*P*-value
Median age (range), years	68 (45–80)	69 (50–80)	65 (45–78)	0.0054
*Sex, n*				
Male	53	29	24	0.69
Female	7	5	2	
*Main tumor location, n*				
Cervix and upper thorax	15	9	6	0.36
Middle thorax	30	15	15	
Lower thorax	14	10	4	
Abdominal	1	0	1	
*ESD-T stage, n*				
T1a	19	11	8	> 0.99
T1b	41	23	18	
*ER-ly, n*				
Positive	22	9	13	0.1
Negative	38	25	13	
*ER-v, n*				
Positive	20	12	8	0.79
Negative	40	22	18	
*ER-HM, n*				
Positive	5	4	1	0.38
Negative	55	30	25	
*ER-VM, n*				
Positive	11	7	4	0.31
Negative	49	27	22	
*Total radiation dose*				
40 Gy		25	–	
50 Gy		9	–	
*Surgery*				
Transthoracic esophagectomy		–	3	
Laparoscopic transhiatal esophagectomy		–	23	

ER, diagnosis by the pathological findings of endoscopic resection specimens; ly, lymphatic invasion; v, vascular invasion; INF, infiltration; HM, horizontal margin; VM, vertical margin

### Treatment outcomes

**Fig. 1:**
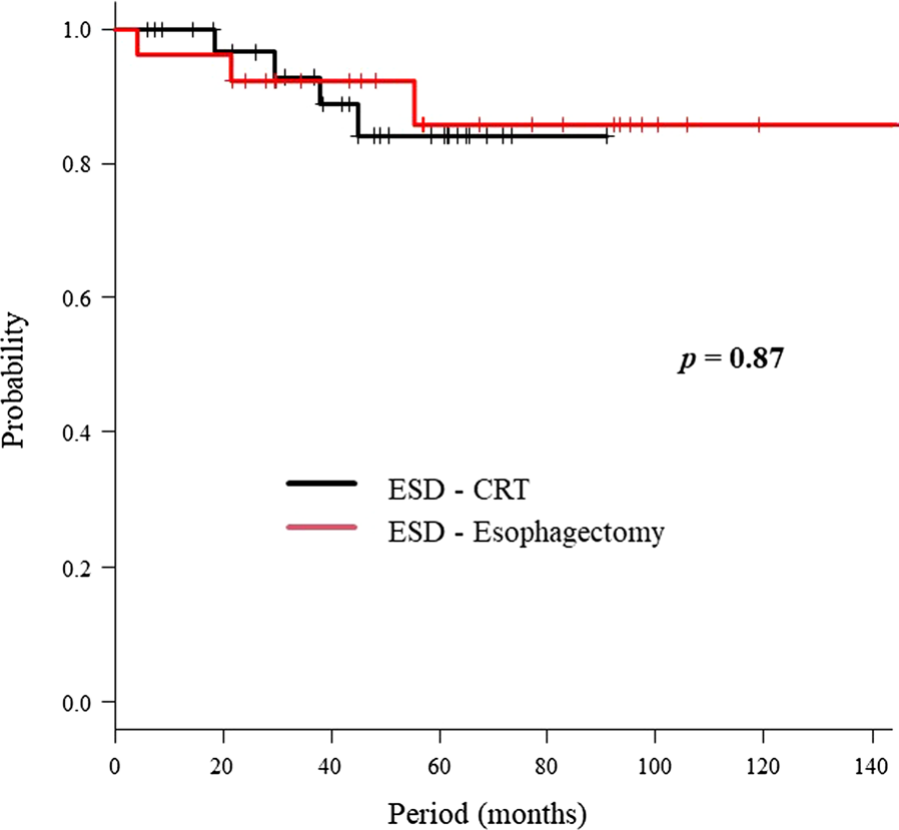
Overall survival rate. The chemoradiation (CRT) and esophagectomy groups had overall survival rates of 84% and 92%, respectively, at 4 years (*p* = 0.87)

Radiotherapy was completed in all patients. Six patients refused to undergo the second chemotherapy cycle. In the CRT group, one patient died of esophageal cancer with distant metastasis, and three died of other causes (colon cancer, liver abscess, and aspiration pneumonitis). In the esophagectomy group, three patients died from other causes (myelodysplastic syndromes, hypopharynx cancer, and pneumonia). The 4-year OS rate of the CRT and esophagectomy groups was 84% and 92%, respectively (Fig. 1); there was no significant difference between the groups (*p* = 0.87).

**Fig. 2:**
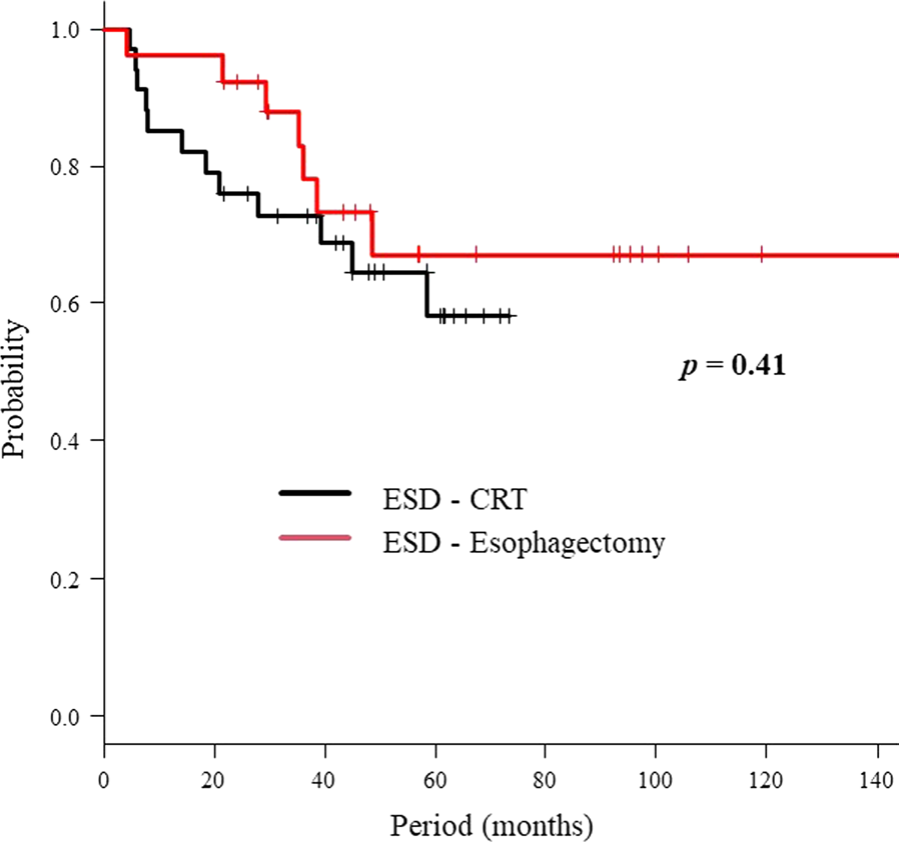
Disease-free survival rate. The CRT and esophagectomy groups had disease-free survival rates of 65% and 73%, respectively, at 4 years (*p* = 0.41)

Tumor recurrence occurred in ten patients in the CRT group. The details of recurrence include local recurrence in six patients (in-field recurrence in three and out-of-field recurrence in three), regional recurrence in one, and distant metastasis in two. All six patients with local recurrence were successfully treated with ESD. In the esophagectomy group, recurrence was observed in five patients, distant metastasis in three, regional recurrence in one, and anastomotic recurrence in one. Table 2 summarizes the patient and clinicopathological characteristics of those with recurrence. The 4-year DFS rate of the CRT and esophagectomy groups was 65% and 73%, respectively; there was not significant difference between the two groups (*p* = 0.41) (Fig. 2).

**Table 2:** Patient and clinicopathological characteristics of 15 recurrent cases

Case	Group	Age	Sex	Tumor location	T stage	Ly	v	HM	VM	Months to disease recurrence (site)
1	CRT	69	Male	Middle thorax	T1b-SM2	(+)	(−)	(−)	(−)	9 (Local)
2	CRT	60	Male	Middle thorax	T1b-SM2	(+)	(−)	(+)	(−)	24 (Distant)
3	CRT	68	Male	Middle thorax	T1b-SM1	(+)	(−)	(−)	(−)	11 (Local)
4	CRT	60	Male	Middle thorax	T1a-MM	(+)	(−)	(−)	(−)	42 (Local)*
5	CRT	80	Male	Lower thorax	T1b-SM2	(+)	(+)	(−)	(+)	32 (Local)*
6	CRT	62	Male	Upper thorax	T1b-SM2	(−)	(+)	(−)	(−)	50 (Local)
7	CRT	71	Male	Middle thorax	T1a-MM	(+)	(+)	(−)	(−)	9 (Local)*
8	CRT	59	Female	Upper thorax	T1b-SM1	(+)	(−)	(−)	(+)	18 (Distant)
9	CRT	73	Male	Middle thorax	T1b-SM2	(+)	(+)	(−)	(−)	9 (Regional LN)
10	CRT	76	Male	Cervix	T1a-MM	(+)	(−)	(−)	(−)	9 (Local)
11	Esophagectomy	78	Male	Lower thorax	T1b-SM2	(+)	(−)	(−)	(−)	29 (Distance)
12	Esophagectomy	58	Male	Middle thorax	T1b-SM1	(+)	(−)	(−)	(−)	38 (Distance)
13	Esophagectomy	70	Male	Middle thorax	T1b-SM2	(+)	(+)	(−)	(−)	48 (Regional LN)
14	Esophagectomy	63	Male	Middle thorax	T1b-SM2	(+)	(−)	(−)	(−)	36 (Regional LN)
15	Esophagectomy	74	Male	Middle thorax	T1a-MM	(+)	(−)	(−)	(−)	35 (Anastomotic)

ly, lymphatic invasion; v, vascular invasion; INF, infiltration; DI, droplet infiltration; HM, horizontal margin; VM, vertical margin; CRT, chemoradiotherapy

*In-field recurrence

### Toxicities

Toxicities in the CRT group were scored according to the National Cancer Institute Common Terminology Criteria for Adverse Events version 4.0. Grade ≥ 3 adverse events (AEs) occurred in 11 (31%) patients, including grade 3 leukopenia in 9 (26%) patients, grade 3 anemia in 2 (6%), and grade 3 esophagitis in 2 (6%). No patients experienced grade ≥ 4 toxicity. Regarding late AEs, grade 2 esophageal strictures were observed in three (9%) patients and grade 3 congestive heart failure in one (3%) during follow-up. No lung-associated AE was observed at grade ≥ 2. Table 3 summarizes the AEs of grade ≥ 2.

**Table 3:** Treatment-related toxicity in the chemoradiotherapy group

	G2 n (%)	G3 n (%)
*Worst grade of hematological parameters during CRT*		
Decreased leucocytes	4 (12%)*	9 (26%)**
Decreased hemoglobin	0	2 (6%)
Decreased platelets	1 (3%)	0
*Nonhematologic acute toxicity*		
Esophagitis, dysphagia	7 (21%)***	2 (6%)
Dermatitis	2 (6%)	0
Diarrhea	1 (3%)	0
*Nonhematologic late toxicity*		
Esophageal strictures	3 (9%)	0
Pericardial/pleural effusion	0	1 (3%)

G, grade; AE, adverse event; CRT, chemoradiotherapy

*Two patients had multiple AEs of G2 (one G2 esophagitis and one G2 decreased platelets)

**Three patients had multiple AEs of G2 or higher (one G3 anemia, one G3 esophagitis, and one G2 esophagitis)

***One patient had multiple AEs of G2 (one G2 diarrhea)

We investigated the safety of surgery in 26 patients in the esophagectomy group. These patients had serious complications, including recurrent nerve palsy (3/26), anastomotic leakage (3/26), respiratory complications (2/26), and gastrointestinal complication (1/26).

## Discussion

With the increase in endoscopic procedures for SESCC, the number of high risk patients with recurrence requiring adjuvant treatment is also expected to increase, and the choice of adjuvant treatment, e.g., esophagectomy or CRT, is becoming a major clinical issue. Compared to surgery, CRT is less invasive, but it also has the drawback of frequent local recurrence. Combined ESD and CRT provide a higher local control rate than definitive CRT alone [18–20], and theoretically fewer cardiopulmonary AEs occur because the appropriate irradiation dose can be delivered after the histopathological findings are confirmed. Our study observed no significant differences in prognosis between the esophagectomy and CRT groups despite the large number of elderly patients in the CRT group. Thus, the safety of CRT was also acceptable.

The efficacy of adding CRT after ESD has been shown in many published reports [2–4, 11, 21]. However, few reports have directly compared the outcomes of surgical treatment and CRT after ESD, and most were small retrospective studies with short follow-up periods [9, 13, 22]. A summary of previous reports comparing the outcomes of surgery and CRT after ESD is shown in Table 4. Some studies did not report follow-up periods, irradiation fields, or disease-free survival rates. We believe that the current study is more clinically informative, with a larger number of cases and a longer follow-up period than previous reports. Tanaka et al. investigated 52 cases of ESD combined with CRT for SESCC with submucosal invasion (19 esophagectomy, 33 CRT) and reported that the 3-year DFS of CRT was comparable to that of surgery (87.4% and 100%, respectively) [9]. Ikeda et al. reviewed 43 patients with clinically suspected SESCC treated with ESD [13], 15 of whom underwent adjuvant surgery, 11 underwent adjuvant CRT/radiotherapy, and 17 were followed up without adjuvant treatments. During the follow-up period of 36 months, the DFS of the adjuvant therapy groups was higher than that of the follow-up group without adjuvant treatment (*p* = 0.04), but there was no significant difference between the adjuvant CRT/radiotherapy and surgery groups (69% and 86%, respectively). Koterazawa et al. investigated 59 patients (28 esophagectomy, 31 CRT) who developed SESCC after noncurative ESD [22]. During a median follow-up of 45 months in the esophagectomy group and 41 months in the CRT group, there were no significant differences (*p* = 0.46) in OS between the two groups. These findings are similar to our findings, and adjuvant CRT after noncurative ESD may be a realistic treatment option for high-risk SESCC.

**Table 4:** Summary of previous reports comparing the outcomes of adjuvant treatment with surgery or CRT after noncurative ESD for SESCC

Authors (reference)	Year	n (CRT/Esophagectomy)	Median follow-up periods (CRT/Esophagectomy)	Basic chemotherapy regimen (5-FU/CDDP)	Irradiation field	DFS rate (CRT/Esophagectomy)
Tanaka et al. [9]	2019	52 (33/19)	NR	700/70	Standard ENI*	(% [at 3 years]) (87.4/100)***
Ikeda et al. [13]	2015	26 (11/15)	43 m/47 m	700/70	NR	(% [at 3 years]) (69**/86)***
Koterazawa et al. [22]	2018	59 (31/28)	41 m/45 m	700/70	Standard ENI*	NR***
Current study		60 (34/26)	46 m/56 m	1000/75	Short ENI	(% [at 4 years]) (65/73)***

CRT, chemoradiation; m, months; ENI, elective nodal irradiation; NR, not reported; DFS, disease-free survival

*Japan Clinical Oncology Group Study JCOG0508 protocol (11)

**Including CRT and RT

***No significant difference between the two groups

A systematic review by Lima [2] reported that patients who underwent ESD followed by CRT/radiotherapy demonstrated recurrence rates ranging from 0 to 27.2% and lymph node recurrence was the most common failure pattern (0–18.2% of patients). In our study, only one (3%) patient had lymph node recurrence, which occurred outside the irradiation field. Although we used a smaller irradiation field than in previous reports [9, 10, 19, 22, 23] to reduce AEs, our clinical results did not appear to be inferior to those of previous reports. One reason for this may be the chemotherapeutic regimen. We used a more potent chemotherapy regimen (5-FU 1000 mg/m^2^ on days 1–4 and 29–32 and CDDP 75 mg/m^2^ on days 1 and 29 [i.e., FP1000/75]) compared to previous studies (5-FU 700 mg/m^2^ on days 1–4 and 29–32 and CDDP 70 mg/m^2^ on days 1 and 29 [i.e., FP700/70]) [9, 10, 19, 22, 23]. Ikawa et al. evaluated 96 patients treated with adjuvant CRT using FP700/70 following ESD for SESCC [23]. Nine (9%) patients developed lymph node recurrence, and the majority of the recurrence involved the elective nodal irradiation field. Tanaka et al. investigated 33 patients with SESCC treated with ESD and CRT [9]. Concurrent chemotherapy was administered in various regimens, with FP700/70 as the basic regimen. No lymph node recurrence was observed in all 9 patients in the high-dose FP (1000/100 or 800/80) group, but it was observed in 4 of 24 (17%) patients in the nonhigh-dose FP group. An intensified chemotherapy regimen may play an important role in controlling potential lymph node metastasis.

Our study suggests that combining reduced field irradiation and intensified chemotherapy (FP1000/75) does not increase the risk of lymph node recurrence outside the irradiation field. In addition, cardiac- and lung-associated AEs at grades ≥ 2 were observed in only one (3%) patient (grade 3 congestive heart failure), which is a low frequency than noted in published studies [10, 11]. In particular, in cases where the primary tumor was located in the middle or lower esophagus, the reduced irradiation field may have provided safety.

In 2018, we published a preliminary report focusing on the feasibility and toxicity of adjuvant CRT after ESD and compared it to the outcomes of adjuvant surgery [12]. However, this study included only squamous cell carcinoma, had a larger sample size (N = 60), longer follow-up (median, 4.9 years), and described more mature toxicity results and clinical data. We concluded that CRT remains an appropriate option for high-risk SESCC treated with ESD. To the best of our knowledge, this is the largest study directly comparing the efficacy of CRT and esophagectomy as adjuvant treatment after ESD.

This study has several limitations, which include its retrospective, single-institution design, and insufficient patient numbers. A multicenter randomized controlled trial is ongoing in China to compare the efficacy and safety of CRT and esophagectomy for high-risk SESCC after ESD [24], and the results are expected shortly.

## Conclusions

This study showed that OS and DFS were not significantly different between the adjuvant CRT and esophagectomy groups, indicating equivalent efficacy in both. Our findings warrant further investigation in the utility of CRT following ESD for patients with high-risk SESCC.

## Data Availability

The dataset from cancer registry can only be acquired under the permission of Kyoto Prefectural University of Medicine. Therefore, we are not able to release the clinical data of this study.

## Abbreviations

AE: Adverse event
CDDP: Cisplatin
CRT: Chemoradiotherapy
CT: Computed tomography
CTV: Clinical target volume
DFS: Disease-free survival
ESD: Endoscopic submucosal dissection
FP: Fluorouracil/cisplatin
FU: Fluorouracil
LVI: Lymphovascular invasion
OS: Overall survival
SESCC: Superficial esophageal squamous cell carcinoma

## References

[1] Al-Kaabi A, Schoon EJ, Deprez PH, Seewald S, Groth S, Giovannini M (2021). Salvage endoscopic resection after definitive chemoradiotherapy for esophageal cancer: a western experience. Gastrointest Endosc.

[2] Wang AY, Hwang JH, Bhatt A, Draganov PV (2021). AGA clinical practice update on surveillance after pathologically curative endoscopic submucosal dissection of early gastrointestinal neoplasia in the United States: commentary. Gastroenterology.

[3] Pimentel-Nunes P, Libânio D, Bastiaansen BAJ, Bhandari P, Bisschops R, Bourke MJ (2015). Endoscopic submucosal dissection for superficial gastrointestinal lesions: European Society of Gastrointestinal Endoscopy (ESGE) guideline—update 2022. Endoscopy.

[4] Ishihara R, Arima M, Iizuka T, Oyama T, Katada C, Kato M (2020). Endoscopic submucosal dissection/endoscopic mucosal resection guidelines for esophageal cancer. Dig Endosc.

[5] Mizumoto T, Hiyama T, Oka S, Yorita N, Kuroki K, Kurihara M (2018). Curative criteria after endoscopic resection for superficial esophageal squamous cell carcinomas. Dig Dis Sci.

[6] Griffin MS, Shaw IH, Dresner SM (2002). Early complications after Ivor Lewis subtotal esophagectomy with two-field lymphadenectomy: risk factors and management. J Am Coll Surg.

[7] Tachibana M, Kinugasa S, Hiroshi Y, Shibakita M, Tonomoto Y, Dhar D (2005). Clinical outcomes of extended esophagectomy with three-field lymph node dissection for esophageal squamous cell carcinoma. Am J Surg.

[8] Suzuki G, Yamazaki H, Aibe N, Masui K, Kimoto T, Shimizu D (2019). Definitive radiotherapy for older patients aged ≥ 75 years with localized esophageal cancer. In Vivo.

[9] Tanaka T, Ueno M, Iizuka T, Hoteya S, Haruta S, Udagawa H (2019). Comparison of long-term outcomes between esophagectomy and chemoradiotherapy after endoscopic resection of submucosal esophageal squamous cell carcinoma. Dis Esophagus.

[10] Hamada K, Ishihara R, Yamasaki Y, Hanaoka N, Yamamoto S, Arao M (2017). Efficacy and safety of endoscopic resection followed by chemoradiotherapy for superficial esophageal squamous cell carcinoma: a retrospective study. Clin Trans Gastroenterol.

[11] Minashi K, Nihei K, Mizusawa J, Takizawa K, Yano T, Ezoe Y (2019). Efficacy of endoscopic resection and selective chemoradiotherapy for stage I esophageal squamous cell carcinoma. Gastroenterology.

[12] Suzuki G, Yamazaki H, Aibe N, Masui K, Sasaki N, Shimizu D (2018). Endoscopic submucosal dissection followed by chemoradiotherapy for superficial esophageal cancer: choice of new approach. Radiat Oncol.

[13] Ikeda A, Hoshi N, Yoshizaki T, Fujishima Y, Ishida T, Morita Y (2015). Endoscopic submucosal dissection (ESD) with additional therapy for superficial esophageal cancer with submucosal invasion. Intern Med.

[14] Nishimura Y, Koike R, Ogawa K, Sasamoto R, Murakami Y, Itoh Y (2012). Clinical practice and outcome of radiotherapy for esophageal cancer between 1999 and 2003: the Japanese Radiation Oncology Study Group (JROSG) survey. Int J Clin Oncol.

[15] Kuwano H, Nishimura Y, Oyama T, Kato H, Kitagawa Y, Kusano M (2015). Guidelines for diagnosis and treatment of carcinoma of the esophagus April 2012 edited by the Japan Esophageal Society. Esophagus.

[16] Akutsu Y, Uesato M, Shuto K, Kono T, Hoshino I, Horibe D (2013). The overall prevalence of metastasis in T1 esophageal squamous cell carcinoma. Ann Surg.

[17] Kanda Y (2013). Investigation of the freely available easy-to-use software ‘EZR’ for medical statistics. Bone Marrow Transplant.

[18] Yoshimizu S, Yoshio T, Ishiyama A, Tsuchida T, Horiuchi Y, Omae M (2018). Long-term outcomes of combined endoscopic resection and chemoradiotherapy for esophageal squamous cell carcinoma with submucosal invasion. Dig Liver Dis.

[19] Kawaguchi G, Sasamoto R, Abe E, Ohta A, Sato H, Tanaka K (2015). The effectiveness of endoscopic submucosal dissection followed by chemoradiotherapy for superficial esophageal cancer. Radiat Oncol.

[20] Suzuki G, Yamazaki H, Aibe N, Masui K, Shimizu D, Kimoto T (2018). Radiotherapy for T1N0M0 esophageal cancer: analyses of the predictive factors and the role of endoscopic submucosal dissection in the local control. Cancers (Basel).

[21] Flor de Lima M, Castro B, Rodríguez-Carrasco M, Libânio D, Pimentel-Nunes P, Sousa O (2022). Best additional management after non-curative endoscopic resection of esophageal squamous cell carcinoma: a systematic review and meta-analysis. Scand J Gastroenterol.

[22] Koterazawa Y, Nakamura T, Oshikiri T, Kanaji S, Tanaka S, Ishida T (2018). A comparison of the clinical outcomes of esophagectomy and chemoradiotherapy after noncurative endoscopic submucosal dissection for esophageal squamous cell carcinoma. Surg Today.

[23] Ikawa T, Ishihara R, Konishi K, Morimoto M, Hirata T, Kanayama N (2019). Failure patterns after adjuvant chemoradiotherapy following endoscopic resection for superficial esophageal squamous cell carcinoma. Cancer Med.

[24] Kam TY, Kountouri M, Roth A, Frossard J-L, Huber O, Mönig S (2018). Endoscopic resection with adjuvant chemo-radiotherapy for superficial esophageal squamous cell carcinoma: a critical review. Crit Rev Oncol Hematol.

